# Seg2Link: an efficient and versatile solution for semi-automatic cell segmentation in 3D image stacks

**DOI:** 10.1038/s41598-023-34232-6

**Published:** 2023-05-22

**Authors:** Chentao Wen, Mami Matsumoto, Masato Sawada, Kazunobu Sawamoto, Koutarou D. Kimura

**Affiliations:** 1 Graduate School of Science, Nagoya City University, Nagoya, Japan; 2 RIKEN Center for Biosystems Dynamics Research, Kobe, Japan; 3 Department of Developmental and Regenerative Neurobiology, Institute of Brain Science, Nagoya City University Graduate School of Medical Sciences, Nagoya, Japan; 4 Division of Neural Development and Regeneration, National Institute for Physiological Sciences, Okazaki, Japan

**Keywords:** Image processing, Neural circuits

## Abstract

Recent advances in microscopy techniques, especially in electron microscopy, are transforming biomedical studies by acquiring large quantities of high-precision 3D cell image stacks. To examine cell morphology and connectivity in organs such as the brain, scientists need to conduct cell segmentation, which extracts individual cell regions of different shapes and sizes from a 3D image. This is challenging due to the indistinct images often encountered in real biomedical research: in many cases, automatic segmentation methods inevitably contain numerous mistakes in the segmentation results, even when using advanced deep learning methods. To analyze 3D cell images effectively, a semi-automated software solution is needed that combines powerful deep learning techniques with the ability to perform post-processing, generate accurate segmentations, and incorporate manual corrections. To address this gap, we developed Seg2Link, which takes deep learning predictions as inputs and use watershed 2D + cross-slice linking to generate more accurate automatic segmentations than previous methods. Additionally, it provides various manual correction tools essential for correcting mistakes in 3D segmentation results. Moreover, our software has been optimized for efficiently processing large 3D images in diverse organisms. Thus, Seg2Link offers an practical solution for scientists to study cell morphology and connectivity in 3D image stacks.

## Introduction

Recent advances in microscopy techniques have enabled scientists to efficiently acquire large-scale and/or high-resolution 3D images with optical and electron microscopy^1–3^. These large 3D images, particularly those obtained through electron microscopy, offer a powerful tool for studying the structure and connectivity of cells in organs, such as brains^4–8^. However, one of the necessary procedures, segmenting cells into individual ones, remains a challenging and time-consuming task^9,10^.

Traditionally, segmenting large 3D cell images requires time-consuming manual annotations^11,12^, which has spurred the development of new automatic segmentation methods^13–17^. The automatic cell segmentation usually involves two main steps. The first step entails the differentiation of individual cells from the background, commonly referred to as semantic segmentation in computer vision literature^18^. The second step involves the discrimination of multiple cells when they are closely attached to each other, known as instance segmentation^18^. Over the years, various deep learning-based techniques have been developed to address these challenges. Some approaches, such as U-Net^14^, focus on semantic segmentation. Although they require other instance segmentation techniques like watershed^19^ to complete the segmentation, they have the advantage of needing less user effort to prepare the training data compared to other deep learning methods for instance segmentation such as StarDist^15^, which aim to solve both semantic and instance segmentation problems using deep learning-based techniques.

Despite the development of these advanced methods, cell segmentation remains a challenging task, and mistakes in segmentation, referring to the differences between automatic segmentation algorithms and human expert judgment, are often unavoidable in real images due to several reasons. Firstly, the segmentation program need to detect cell boundaries to separate closely attached cells, which is often challenging in real images. For instance, fluorescence microscopy images may contain cells attached to each other without clear boundaries (Supplementary Fig. S1). On the other hand, although cell boundaries could be visible in electron microscopy images, they may be absent in some regions (Supplementary Fig. S1). Moreover, due to the specific nature of 3D segmentation tasks, mistakes in a single slice can easily propagate to other slices, necessitating a substantial amount of manual correction by users (Supplementary Fig. S1). The complexity of cell shapes, such as those of neurons, further exacerbates the challenge of detecting cell boundaries. As a result, even advanced methods are prone to mistakes and require manual corrections based on experts’ knowledge and experience, as demonstrated by a previous study^20^. Secondly, many real images, such as those obtained through electron microscopy, may exhibit slice displacement along the z-axis, making automatic alignment challenging, particularly when non-rigid displacement occurs. Such displacements can cause incorrect linking across slices, leading to erroneous segmentation results that require manual corrections (Supplementary Fig. S1).

For the reasons stated above, we consider that automated segmentation methods are unlikely to reach perfection and will continue to require manual corrections to reconcile the differences between automatic and human judgments for the foreseeable future. Therefore, if we can develop a semi-automatic software program that combines automatic segmentation and computer-assisted manual correction while maximizing operational efficiency, it will substantially help scientists to quickly and accurately segment 3D images. The following features would be ideal in such a software program. (1) The ability to automatically generate instance segmentation results, which is required for post-processing the semantic segmentation result by techniques such as U-Net. (2) Efficient correction of mistakes in the instance segmentation results on a slice-by-slice basis, with the corrected results used to generate more accurate segmentation in surrounding slices, thereby reducing the amount of manual correction required. (3) Optimized procedures for manual corrections, as well as optimized computational and storage efficiency to reduce user operation time and hardware requirements. Previous software programs for assisting 3D cell segmentation either could not utilize the semantic segmentation results as input, generate less accurate instance segmentation results, or lack key functions essential for manual corrections of the segmentation mistakes^11,12,21–24^.

Here we developed Seg2Link, a semi-automatic segmentation software that can take semantic segmentation results (cellular/non-cellular predictions) as inputs, performs automatic instance segmentation using watershed 2D (in *x–y* plane) + cross-slice linking (along *z* axis) which is more accurate than the watershed 3D used by other software, or it can directly take instance segmentation results as inputs. Seg2Link can segment entire images as well as user-defined regions. Additionally, our software program allows users to efficiently perform computer-assisted corrections of segmentation results through techniques such as utilizing corrected segmentation results in adjacent slices, quick localization of cells, multiple-step undo/redo functionality, various optimized tools for inspecting and correcting mistakes, and more, which are often not available in existing software. We consider Seg2Link successfully integrates many of the key functions required by semi-automatic 3D cell segmentation and can help scientists in analyzing the cell morphology and connectivity in organs more efficiently.

## Results

### Overview

**Figure 1:**
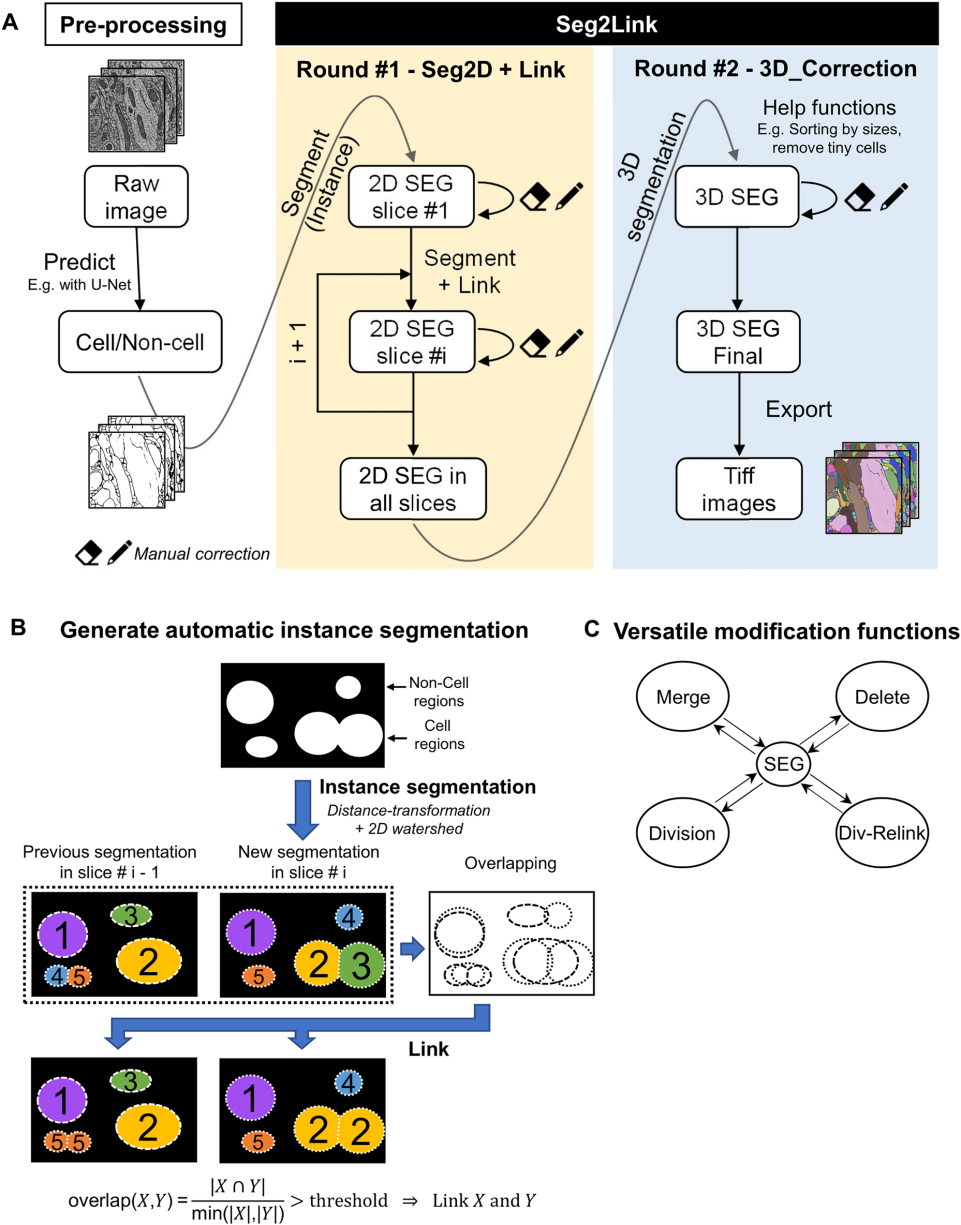
Seg2Link workflow and core functions. (**A**) Seg2Link workflow. (**B**) The methods for automatic segmentation. (**C**) The manual modification functions. SEG is an abbreviation for segmentation.

Seg2Link is made up of two modules (Fig. 1A). The first module, Seg2D + Link, takes cellular/non-cellular predictions on each pixel generated by automatic semantic segmentation techniques as input and automatically performs instance segmentation to divide the cell regions into individual cells, each with a specific numeric ID, in each slice. It then links the cells with those in previous slices along the *z*-axis (Fig. 1A, left and middle panels). Users can manually correct the segmentation results immediately after the automatic segmentation in each slice, so that to help the software improve the segmentation quality in subsequent slices. After all the slices have been segmented and corrected, the instance segmentation results in the entire 3D space can be exported and imported into the second module, 3D Correction, which allows the user to comprehensively check and correct any remaining mistakes in each 3D-segmented cell (Fig. 1A, right panel). The 3D Correction module can also take the instance segmentation results generated by other automatic methods as inputs. The final corrected 3D segmentation results can be exported as image sequence files for further analyses in other software.

We created a set of graphical user interfaces (GUI) using the napari library^25^ to allow users to specify images and parameters, and perform semi-automatic segmentation. We have developed essential functions such as cell merge, division, cross-slice linking, and undo/redo in 3D space, which are not built-in functions of the napari library. Users can use the tool in napari to check and edit the labels in the results and can use our pre-defined hotkeys and GUI buttons to perform automatic segmentation and manual corrections (see next section).

### Semi-automatic segmentation

This software's core module, Seg2D + Link, allows users to perform rapid slice-by-slice automatic instance segmentation and manual corrections. Users can generate cellular/non-cellular predictions using other programs with deep learning-based semantic segmentation techniques (We provided one—see “Methods”), which can then be exported as TIFF images and used as inputs to the Seg2D + Link module. In the automatic instance segmentation part, the program applies a 2D watershed^19^ to the cellular/non-cellular predictions by a deep neural network and generates segmented cell regions in each slice (Fig. 1B). From the second slice, our program automatically links the segmented cells with cells in previous slices along the z axis using the overlap linking method (Fig. 1B). The software allows users to freely correct the mistakes in the automatic instance segmentation results using various commands (Fig. 1C). Users can also apply multi-step undo and redo functions to user operations to quickly go back to the previous states in case a misjudgment occurs, which is not a built-in function in the napari platform and is not supported by many of the 3D segmentation software. Our software also automatically saves the segmentation results in every slice to the hard disk, allowing the user to resume the previous results later. We also accelerated the modification and the caching/saving processes by designing a custom data structure (Supplementary Fig. S2 and Table S1—see “Methods” for details). During our testing, the automatic segmentation + manual correction takes ~ 3 min per slice on the first 10 slices of the demo dataset with ~ 700 cells per slice, using a laptop computer (See “Methods”). Specifically, manually correcting the first slice (without automatic linking) took 8 min, while manually correcting each subsequent slice (with automatic linking) from #2 took only ~ 2 min, indicating a substantial increase in segmentation accuracy by utilizing the corrected segmentation results in previous slices with our software.

**Figure 2:**
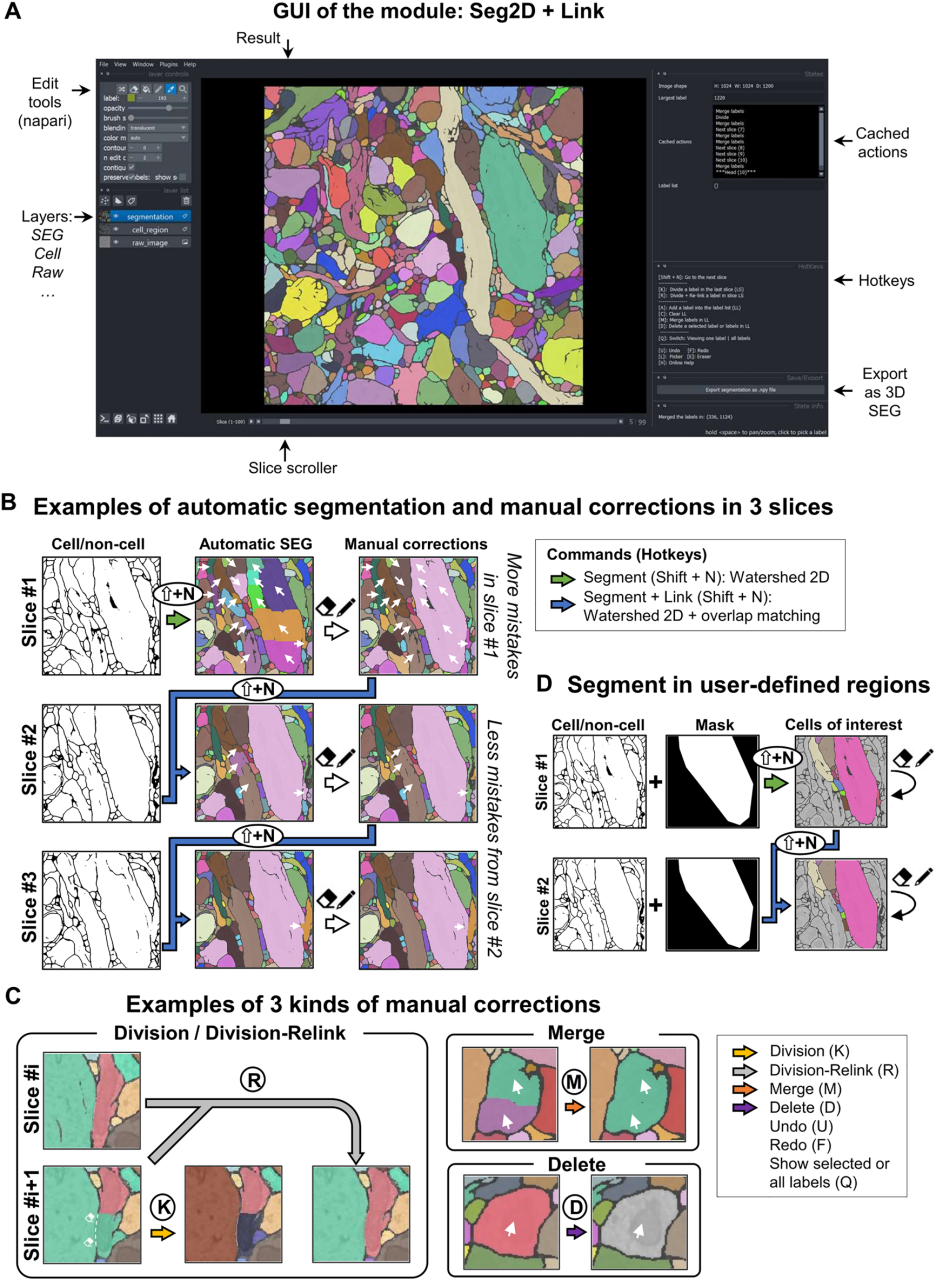
Functions of the Seg2D + Link module. (**A**) The GUI of the main window. (**B**) The process of semi-automatic segmentation in slices #1, #2, and #3. Different colors indicate different cells. The arrows on the images indicate the regions with mistakes in the automatic segmentation and the results after manual corrections. (**C**) The manual correction functions. Arrows on the images indicate the regions to be corrected. (**D**) The masking function for segmentation in user-defined regions.

The main window of the Seg2D + Link module displays the current segmentation results, as well as raw and prediction images in different layers (Fig. 2A). The automatic segmentation and the manual correction functions in Seg2D + Link were carefully designed so that users can easily perform the segmentation. First, 2D watershed + link typically produces good segmentation results with few mistakes. Although the segmentation in the first slice frequently contains a few over-segmented areas, they are easily corrected with the merge command described in Fig. 1C, and the manually performed merge will guide the program to automatically merge the over-segmented areas in the next slice using the overlap linking algorithm, which greatly improves the segmentation quality of the following slices (Supplementary Fig. S3). As a result, users typically only need to make few manual corrections in the slice #2 and afterwards (Fig. 2B). Additionally, merge/delete operations performed on any subsequent slice are also automatically applied to previous slices to improve operational efficiency. Secondly, Seg2Link's correction function requires very few manual operations to perform. For example, relinking cells after division requires only editing the cell boundary in slice # i and then pressing the hotkey R to finish the division and relinking with the previous slice # i-1 (Fig. 2C). Merging or deleting cells only requires a mouse click and pressing the hotkey A to select each cell and add it to the list, followed by a press of M or D to complete the merging or deleting (Fig. 2C). Furthermore, when users are only interested in a specific subregion of a 3D image, Seg2Link allows them to specify the subregion with a mask image and segment the specified region selectively (Fig. 2D).

### Comprehensive inspection and correction

**Figure 3:**
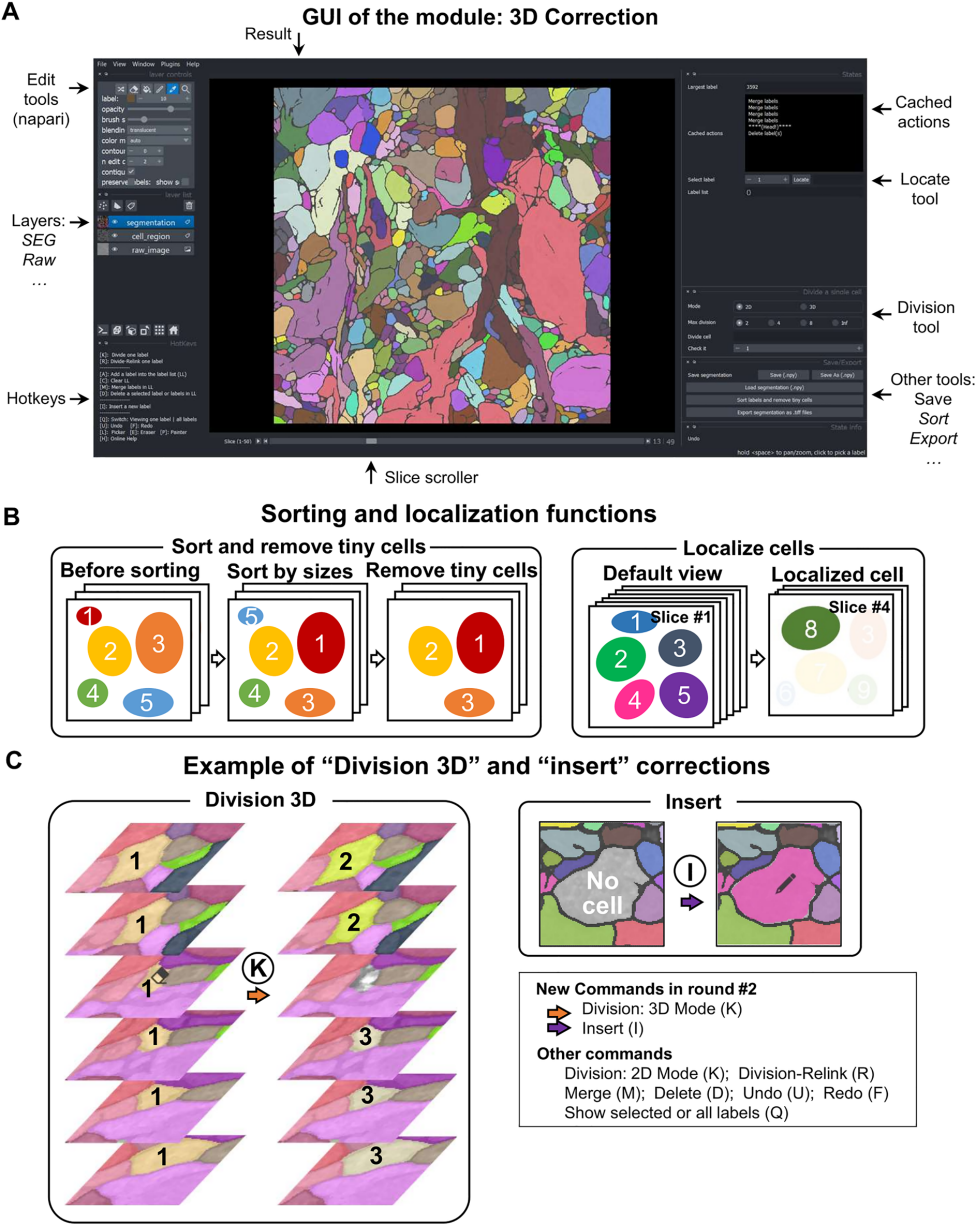
Functions of the 3D correction module. (**A**) The GUI of the main window. (**B**) The cell sorting, cell removal, and cell localization functions. (**C**) The manual correction functions. The numbers 1, 2, and 3 indicate the cell IDs before and after 3D division. The eraser and pen on the images indicate the cleared cell region and the newly inserted cell region, respectively.

The second module, 3D correction, allows the user to thoroughly check and correct the 3D segmentation results obtained from the Seg2D + Link module to confirm that each cell is correctly segmented as a 3D instance (Fig. 3A). It can also take instance segmentation results generated from other techniques as inputs. We designed two functions to make it easier for users to inspect the segmentation results in the entire 3D image. First, large cells are potentially more important, but inspecting all large cells in a 3D image with thousands of cells is difficult. Our software allows users to sort 3D cells by volume size, which is the measure of space each cell occupies in three-dimensional space, so that users can inspect the cells from largest to smallest based on their IDs. It also allows users to remove cells that are smaller than a user-defined threshold (Fig. 3B, left), which are often not of interest to users. Second, because a single cell typically occupies a few slices which is a small portion of all slices, visually searching for a cell with a specific ID among thousands of slices is time-consuming and boring. Our software allows users to quickly locate a specific cell and jump to the middle slice of it (Fig. 3B, right). With these two functions, users can quickly find and inspect cells of interest.

Aside from the two inspection functions mentioned above, we also added two manual correction functions that are needed for the 3D correction module but not appropriate to perform in the Seg2D + Link module. First, instead of dividing a cell in each 2D slice, users can divide it in 3D space, which is useful for separating two cells that are incorrectly linked along the z-axis (Fig. 3C, left). Second, users can insert new cells that have not been detected by deep learning predictions (Fig. 3C, right).

In addition, we improved the computational efficiency of cell localization, which is critical because all implementations of manual corrections require the cell to be localized first. Since the time required for localization increases with the 3D image size, computational efficiency depends on the entire image size rather than the size of the cells of interest, which can become a serious problem. We speed up this function by storing each cell's bounding box (bbox) information in a cache (Supplementary Fig. S4), so that the software can search for a cell in a much smaller subregion, and the time required for localization is primarily determined by cell size rather than the image size. During our tests on a larger dataset than the demo dataset (see “Methods”), we randomly selected 1/50 of the 52, 237 segmented cells and found that localizing a cell without using bbox takes 2.87 secs on average (5th and 95th percentile: [2.84, 2.92]), whereas localizing a cell using bbox takes 0.028 secs on average (5th and 95th percentile: [0.00, 0.09]), indicating a 103-times acceleration.

### Compare our watershed 2D + Link with watershed 3D in segmenting cell/non-cell regions

**Figure 4:**
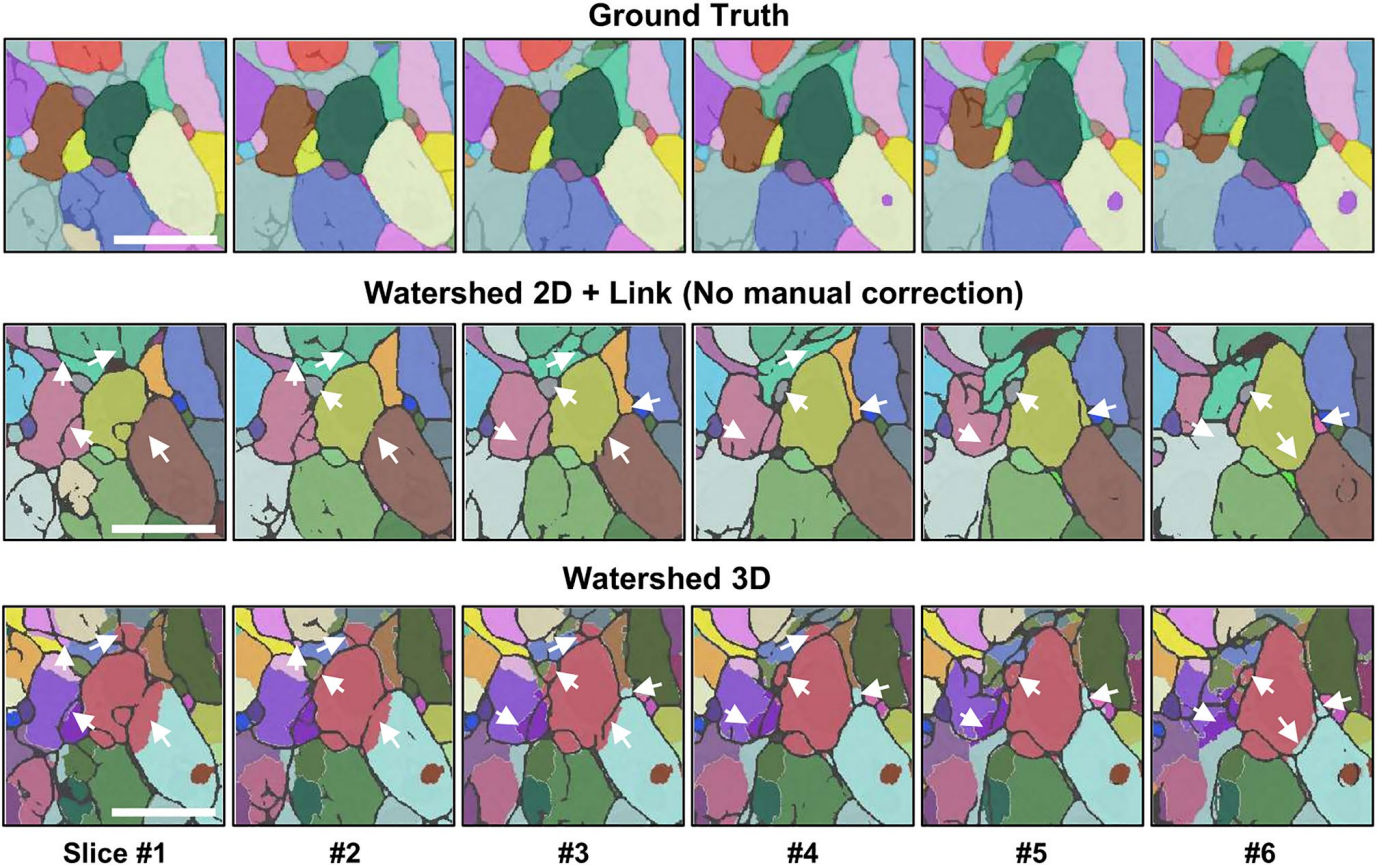
The automatic segmentation results with two different approaches. (Top) The ground truth indicating the correct segmentation results. (Middle) Segment cells with 2D watershed + overlap linking using Seg2D + Link, without performing manual correction. (Bottom) Segment cells with 3D watershed using the “Distance Transform Watershed 3D” function in the plugin MorphoLibJ in ImageJ^26^. Scale bars: 1 µm. The arrows indicate the same regions with correct (watershed 2D + link and ground truth) and incorrect (Watershed 3D) cell boundaries. The results shown here are from the EM demo dataset. We only show a part of the segmentation in the first six slices here. For the comparison results in larger regions, see Table 1 and Supplementary Fig. S5.

When users have obtained the semantic segmentation results (cell/non-cell regions) and need to transform them into individual cells, one popular way is watershed 3D which is available in other software^24,26^. We chose the watershed 2D + Link approach because it is superior to watershed 3D in following aspects: First, our results on the EM demo dataset show that 3D watershed can produce more boundary mistakes than 2D watershed + overlap linking (Fig. 4 and Supplementary Fig. S5), likely because cell boundaries in each x–y slice are inferred with 3D watershed using boundaries in neighboring x–y slices along the z-axis while the resolution along z-axis is typically lower than that in the x–y plane. Correcting such incorrect boundaries requires users to manually paint many pixels, increasing the time cost. In addition, the watershed 3D also generates more mistakes of under-segmentation and over-segmentation, which again requires additional time for correction (Table 1). Second, our 3D Correction module requires much more time than Seg2D + Link to cache/save intermediate results (Supplementary Table S2), which further slows down the corrections of the watershed 3D results with more segmentation mistakes. Finally, the 3D watershed is computationally expensive and cannot handle large images on a typical personal computer. In our experiment, processing the entire EM demo dataset with 1200 slices using the MorphoLibJ plugin’s 3D watershed^26^ required 44 GB of memory, while our software’s Seg2D + Link and 3D correction modules processed the same dataset with only 0.7 GB and 2.7 GB, respectively. For these reasons, we recommend users follow the default workflow (i.e., Seg2D + Link), especially when processing large image stacks with complex cell shapes.

**Table 1:** The comparison of different types of mistakes generated by watershed 2D + Link or by watershed 3D from the EM demo dataset in slice # 1 and 2. The boundary mistakes were manually counted (see Supplementary Fig. S5). The under-segmentation and over-segmentation mistakes were defined as the ratio of cells in ground truth images with mistakes, calculated automatically with a python program using the ground truth data.

Methods	Boundary mistakes		Under-segmentation		Over-segmentation	
	Slice #1	#2	#1	#2	#1	#2
Watershed 2D + Link	0	0	45%	46%	42%	35%
Watershed 3D	67	69	99%	97%	71%	72%

### Segmentation results of various datasets

**Figure 5:**
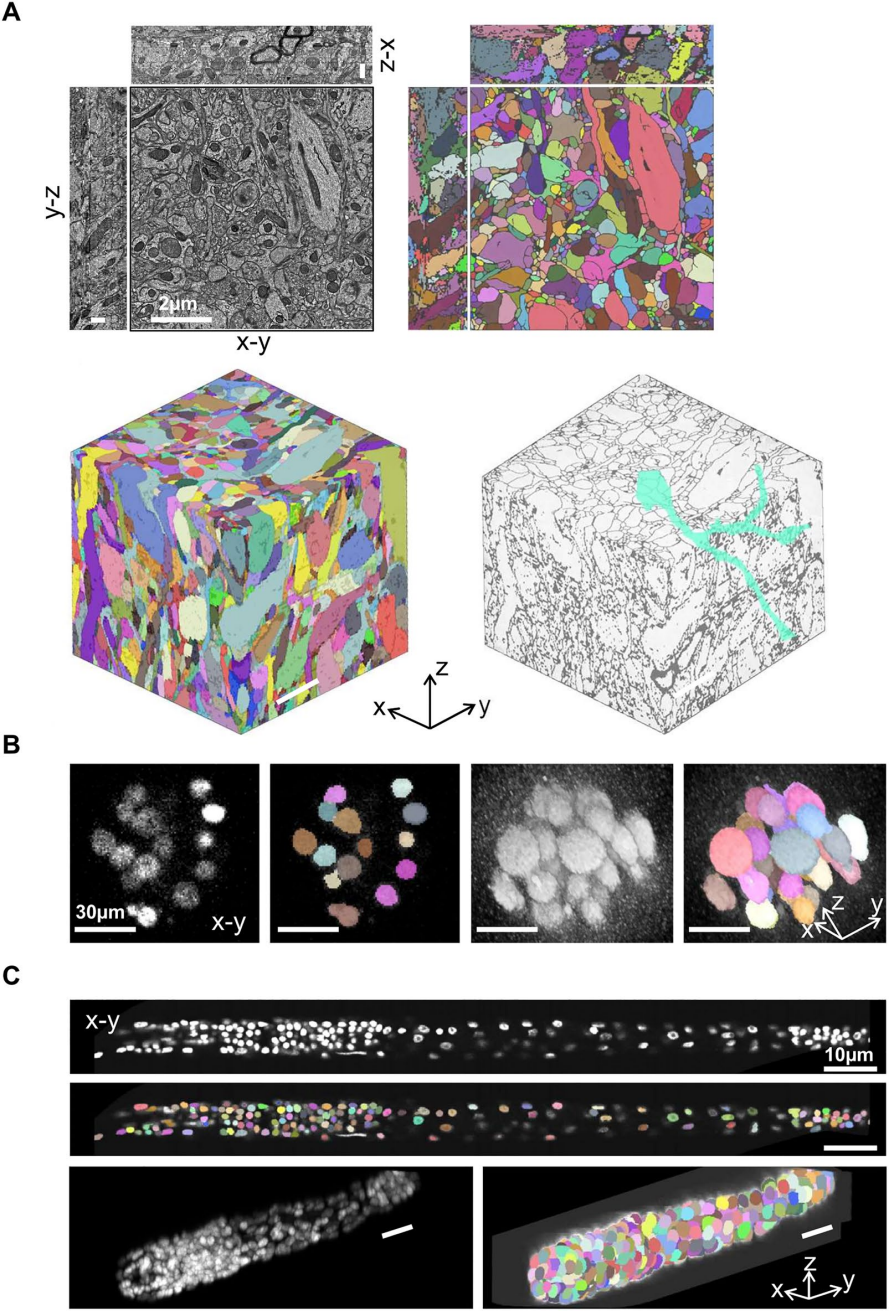
The examples of segmentation results in three datasets. (**A**) Results of the mouse brain dataset. Top: The orthogonal view of the raw image (left) and the segmentation result (right) in slices #1–50. Bottom: The 3D view of the slices #1–50 demonstrating all segmented cells (left) and the morphology of a specific cell (right). All scale bars: 2 µm. (**B**) Results of the mouse embryonic cells dataset. Panels from left to right are 2D view of the raw image and the segmentation result of slice #23, 3D views of the raw image and the segmentation result. All scale bars: 30 µm. (**C**) Results of the *C. elegans* cells dataset. Panels from top to bottom, left to right are 2D view of the raw image and the segmentation result of slice #20, 3D views of the raw image and the segmentation result. All scale bars: 10 µm.

To demonstrate the broad applicability of our software, we present the segmentation results obtained from three distinct datasets, including the mice brain dataset via electron microscope (Fig. 5A), an embryonic mice cells dataset^27^ (Fig. 5B) and a post-embryonic *C. elegans* larva cells dataset^28^ via optical microscope (Fig. 5C). The results indicate that our software is effective in segmenting cells from image stacks captured via diverse imaging techniques, in different organs. Further details regarding these datasets can be found in the Methods section.

## Discussion

Automatic segmentation of 3D cellular images is a challenging task. Instead of improving the automatic method, we created a semi-automatic solution called Seg2Link, which uses deep learning predictions as input and assists users in quickly transforming imprecise predictions into a precise instance segmentation by providing an easy-to-use GUI as well as rich computer-assisted correction functions. Furthermore, we optimized Seg2Link’s data structure and algorithm, allowing users to efficiently segment large 3D cell images with billions of voxels.

To segment cells in high-resolution 3D image stacks, mistakes generated by the automatic segmentation need to be corrected by experts, and it is preferable for such corrections to be performed quickly in a typical personal computer. In this research, we suggested following approaches to address these challenges: (1) We use watershed 2D + cross-slice linking for the automatic instance segmentation (Fig. 1A and B), which is more accurate and computationally efficient than watershed 3D (Fig. 4, Table 1 and Supplementary Fig. S5). (2) We enable users to interactively correct the segmentation in each slice and update the segmentation results in the adjacent slices using the corrected results, thus reducing the amount of required corrections (Fig. 2B and Supplementary Fig. S3). (3) We designed specific data structures in our software program, such as label lists in the Seg2D + Link module and bounding boxes in the 3D correction module, which speed up the computations underlying correction and localization functions, as well as caching processes (Supplementary Fig. S2, S4, Table S1, S2 and “Methods”). (4) We designed various functions such like undo/redo, cell merge/delete/division/division + relink, cell sorting/localization, and so on, which are essential for the computer-assisted corrections and can be performed easily using hot-keys (Figs. 2 and 3). Our software combines powerful deep learning techniques with post-processing capabilities and efficient mistake correction, making deep learning-based semi-automated segmentation a realistic option.

In the past, other software programs have made significant contributions to advancing the field of 3D segmentation^21–24,29,30^. It is beneficial to compare the efficiency between our software with them. However, we found that these programs lack essential features for manually correcting 3D segmentations, making such a comparison difficult and possibly unnecessary (Table 2). In terms of functionality, one of the closest competitors, UNI-EM^24^, uses the less accurate watershed 3D method (Table 1), and it has several flaws that make accurate manual correction difficult, such as the inability to merge cells from different slices and frequent failure in cell division and color display functions. While NeuTu^23^ claims to offer extensive manual correction features for large 3D segmentation, its design is primarily geared toward internal use by Janelia research center and their collaborators, making it less accessible to external users. VAST Lite^22^, on the other hand, may be better suited for manual segmentation from scratch but may not be the best choice for corrections, since it lacks essential functions such as the ability to freely divide a cell. Other software programs^21,29,30^ lack manual correction functions or had substantially limited capabilities.

**Table 2:** Comparison of 3D segmentation software features. ✓: has the feature. △: partially has the feature. –: does not have the feature.

	Automatic segmentation			Manual correction/inspection								Multiple platform	Open source
	Predict cell/noncell regions	Segment cell regions into individual cells	Mask	Merge	Delete	Division (2D)	Relink after division	Division (3D)	Undo/redo	Sort by sizes / Remove tiny	Locate cells		
Ilastik^29^	✓	✓ watershed 3D	–	–	–	–	–	–	–	–	–	✓	✓
TrackMate^30^	–	△ 2D SEG (imported from other software) + Link	–	–	–	–	–	–	–	–	–	✓	✓
Dojo^21^	–	–	–	△ but only within the same slice	✓	✓	–	–	✓	–	–	△ not in Windows	✓
UNI–EM^24^	✓	✓ watershed 3D	–	△ but only within the same slice	✓	✓	–	–	✓	–	–	△ not in macOS	✓
NeuTu^23^	–	–	–	✓	✓	–	–	✓ requires adding seeds	△ cannot undo after confirming a merge	–	✓	△ not in Windows	✓
VAST Lite^22^	–	–	–	✓	✓	△ but only with straight line	–	–	–	–	✓	–	–
Seg2Link	–	✓ watershed 2D + Link	✓	✓	✓	✓	✓	✓ requires annotating boundary	✓ multiple steps	✓	✓	✓	✓

While Seg2Link is highly efficient in processing 3D images with billions of voxels, it has limitations when processing even larger 3D images: The 3D correction module cannot process 3D segmentation exceeding the memory capacity (E.g., an 16-bit segmentation results with 4,000 × 4,000 × 1,000 = 16 billion voxels roughly occupies 32 GB memory), which is a desired feature available in some other software for analyzing huge datasets such as entire brains^22,23^. This issue may be solved in the future by adding additional functions allowing users to divide the entire image into smaller sub-images to segment them separately, and then combine the results later. It is worth noting that many biomedical studies focus on a small portion of an organ, resulting in 3D optical/electron microscopy images that can be processed within memory limits.

Apart from our excellent design for performing quick and easy segmentation/correction, the advantages of Seg2Link are also drawn from existing data processing tools developed by the scientific computing/image processing communities. These tools allowed us to write a concise program implementing both the upper-level visualization and the underlying computation. The recently released napari library, for example, includes a GUI for viewing and editing various types of image stacks. It also allows us to add new widgets and hotkeys to perform custom functions. NumPy, SciPy, and scikit-image are array/image processing libraries that allow us to write image processing functions. Dask, Python's big data processing library, enables our software to dynamically load files on disk, reducing memory usage.

In summary, Seg2Link can be used for a wide range of 3D biomedical images. Typical application scenarios include: analyzing the morphology and connectivity of neurons in the brain (Fig. 5A) to better understand the mechanisms underlying their development and functions^31^, studying the cell morphology and spatial distribution of embryonic^27^ (Fig. 5B) or post-embryonic organisms^28^ (Fig. 5C) to gain insights into developmental mechanisms, and analyzing 3D cultured cells, which is of great significance for disease modeling and drug discovery^32^.

## Methods

### Computational environment

Seg2Link is entirely CPU-based and does not require a GPU. All of the analyses about the runtime in this manuscript were carried out on a laptop computer running Windows 11 with the CPU AMD Ryzen 9 5900HS and 32 GB RAM. We also confirmed that the software can run on other desktop/laptop computers with Windows, macOS, or Linux systems. Seg2Link relies on napari, Dask, NumPy, SciPy, scikit-image, and other Python libraries for visualization and underlying computation. Users can easily install Seg2Link and its dependencies using the pip command.

### Image dataset

To demonstrate the functions of our software, we used a portion (1024 × 1024 × 1200 voxels) of a 3D EM image dataset (minnie65_8 × 8 × 40) of the visual cortex in mouse brain, which is publicly available (https://bossdb.org/project/microns-minnie). The dataset contains raw images as well as segmentation results with proofreading, with an x–y plane resolution of 8 nm/pixel and steps of 40 nm between slices. In 12 of the 1200 slices (i.e., slices # 50, 150, …, 1150), we transformed the provided segmentations to cell/non-cell regions as the ground truth for training a 2D U-Net model. The trained 2D U-Net was then used to predict cell/non-cell regions across all 1200 slices of the 3D image. These predictions and raw images (stored as 2D TIFF image sequence) were then imported into Seg2Link to perform the semi-automatic segmentation. We also used a larger portion (2048 × 2048 × 1200 voxels) of the same dataset to test the localization time with and without using bbox.

In addition, we used two other datasets for demonstrating the segmentation results of optical cell images using our software. The first dataset includes cells of mouse embryo^27^. It is a 3D image (Emb1_t501.tif) taken by an inverted widefield microscope and is publicly available in the image set BBBC050 from the Broad Bioimage Benchmark Collection^33^ at https://bbbc.broadinstitute.org/BBBC050. This dataset has the size of 112 × 114 × 51 voxels, with an *x*–*y* plane resolution of 0.8 µm/pixel and steps of 2.0 µm between slices. The second dataset includes cells of *C. elegans* at the L1 stage^28^, which is a 3D image taken by a confocal microscope and is publicly available at https://doi.org/10.5281/zenodo.5942574 (C18G1_2L1_1.tif). This dataset has the size of 1244 × 140 × 64 voxels, with an *x*–*y* plane resolution of 0.116 µm/pixel and steps of 0.122 µm between slices.

### Architectures of Seg2Link

#### *Module 1: Seg2D* + *Link—Automatic segmentation*

The automatic segmentation part of the Seg2D + Link module processes each slice of the 3D image one by one (Fig. 1A). The processing of every single slice consists of two sequentially executed steps: 2D segmentation and cross-slice linking (Fig. 1B).

The 2D segmentation procedure uses distance transformation to convert each slice of cellular/noncellular predictions into a distance map, finds local maximums as seeds, and then applies 2D watershed^19^ to segment the image in the current working slice into individual cells (Fig. 1B). To mitigate over-segmentation, Seg2D + Link applies a Gaussian blur to the converted distance map and then uses the h-maxima transform^34^ to filter the multiple local maxima within the same cell. By default, Seg2Link segments the entire 2D image in each slice. When the user provides a mask image indicating the region of interest (ROI), the program calculates the proportion of each segmented cell falling within the ROI. Cells with this proportion less than a user-specified threshold (default value 0.8) are deleted automatically (Fig. 2D).

From the second slice, the program will automatically link the segmented cells to the previous slice. For each pair of cells in the two adjacent slices, the program computes the overlap coefficient^35^: Suppose there is a cell X in slice *i* and a partially overlapping cell Y in slice *i*−1. Their overlap coefficient is calculated using the equation below:

1 $$overlap\left(X,Y\right)=\frac{area(X\cap Y)}{\mathrm{min}[area\left(X\right), area\left(Y\right)]}$$

The calculated overlap coefficient is compared to a user-specified threshold (default is 0.5). When the overlap exceeds the threshold, the program will link X and Y to form a single cell (Fig. 1B).

#### *Module 1: Seg2D* + *Link—Manual correction*

Following the automatic segmentation and linking of each slice, the user can make the four types of corrections: 1. Merge multiple cells into a single cell; 2. Delete one or more cells; 3. Divide a cell in the current working slice into multiple cells; 4. Divide a cell in the current working slice and relink the results to the previous slices (Fig. 2C). Because of the specific data structure we used in the Seg2D + Link module to accelerate the modifications/caching/saving (see below), operations 1 and 2 can be applied to cells in any completed slice, whereas operations 3 and 4 can only be applied to cells in the current working slice. In module 2, 3D correction, users will be able to apply division and division-relink more freely in any slice (see below).

After executing each operation, our program will cache/save the current segmentation status in memory/hard disk. If users realize that they made incorrect operations later, they can easily return to previous states using the undo/redo function (up to 10 steps by default, but users can modify it). If users quit the software, they can also easily restart from the point of interruption (but the previous states for undo/redo will be cleared).

Seg2Link makes use of the napari viewer to let users view and edit segmentation results (Fig. 2A). Users can pan and zoom the images to examine different regions in detail. The reference images and segmentation results can be overlaid to aid in visual inspection. The napari editing tools allow the user to select cells, and paint or correct their cell boundaries. In conjunction with our correction programs, these napari functions could assist users in quickly correcting the 3D segmentation results.

#### *Module 1: Seg2D* + *Link—Underlying data structure*

We designed a custom data structure for Seg2D + Link: The 3D segmentation result is saved as a series of 2D segmentation results corresponding to each slice. In each slice, the 2D segmentation is represented by a 2D label image (a 2D array) that stores the original 2D segmentation results from the 2D watershed, and a label list (a 1D list) that originally stores labels in the 2D segmentation result and will be updated when labels change due to link/merge/delete, etc. (supplementary Fig. S2). With this design, the program only needs to modify/cache/store a much smaller data structure: a 2D array + a group of 1D lists, rather than the large 3D array (supplementary Table S1). As a result, we are able to perform the modification and cache/save the intermediate state at a faster rate while using less memory/disk space.

While this custom data structure speeds up the functions, it requires an additional calculation process to display the updated segmentation results based on the original 2D segmentation results and the updated label lists. When there are too many slices to display, the computation becomes time-consuming. To reduce computational load, Seg2Link only displays segmentation results in a limited number of slices surrounding the current working slice (by default, 100 slices).

#### Module 2: 3D correction—Manual correction

The segmentation results from module 1 can be saved as a 3D array (npy format) and imported into module 2 to correct any remaining mistakes. The 3D segmentation results (2D TIFF image sequence) exported from other software can also be imported into module 2 for correction (e.g., The watershed 3D segmentation results shown in Fig. 4 were obtained using a different program and imported to the Module 3D correction). After completing all necessary corrections in module 2, the user can export the final segmentation results as 2D TIFF image sequence for further analysis in other software (Fig. 1A).

Module 2 offers the same four manual corrections as module 1: merge, delete, division, and division-relink. In contrast to module 1, the division and division-relink functions in this module can be conveniently implemented at any slice, but at the cost of a slower rate for modifications/caching/saving and increased memory/disk space requirements (See below).

In addition to the division and division-relink functions which are applied to a 2D subregion of a cell in a specific slice, we add a 3D division function in module 2 for dividing a cell in 3D space. We also add a function to insert a new cell that was not detected by deep learning, requiring users to manually paint the cell region with an automatically assigned new label (Fig. 3C).

#### Module 2: 3D correction—Easy inspection

Module 2 was designed to allow users to easily inspect the segmentation results. It allows users to do the following: 1. Sort cell ID numbers by the volume size in descending order, so that large cells that may be more important can be easily selected and checked. 2. Remove cells smaller than a user-defined threshold, so that cells that are potentially irrelevant can be ignored. 3. Select a cell ID and jump to the central slice of the cell, which is useful when searching a cell through thousands of slices (Fig. 3B).

#### Module 2: 3D correction—Underlying data structure

In module 2, it is necessary to display the entire 3D segmentation results so that users can perform comprehensive visual inspections. The data structure in module 1 is no longer appropriate because it requires additional computations to display the updated segmentation results, which is time-consuming now. Instead, we use a simple data structure, a 3D array, to store the segmentation results (Supplementary Fig. S4).

One critical issue about using a 3D array as data structure is that searching for a cell's location takes a long time due to the large search space. Seg2Link solved this problem by pre-calculating and storing the bounding boxes (bbox) of all cells in memory (Supplementary Fig. S4). Following each correction operation, the bbox of relevant cells are updated. In this way, the program can search for a cell in a much smaller sub-region and with much less time.

Another issue is that caching the entire 3D array is very space-intensive, making the undo/redo functions impractical. Our program solved this problem by caching changes only in the sub-region with modifications, which requires much less memory (up to 5 steps of undo/redo is possible by default, but users can modify this).

## Supplementary Information

Supplementary Information.

## Data Availability

The Seg2Link source code and user guide for installing and using the software can be found at: https://github.com/WenChentao/Seg2Link. We have also provided a program for training a 2D U-Net to predict cell/non-cell regions, which can be found at: https://github.com/WenChentao/seg2link_unet2d. In addition, the EM demo dataset, including raw images and cell/non-cell predictions by the trained 2D U-Net, is available for download at: https://osf.io/wngty/.
