# Supplementary material for: Seg2Link: an efficient and versatile solution for semi-automatic cell segmentation in 3D image stacks
Source: Sci Rep. 2023 May 22;13:7109. doi: 10.1038/s41598-023-34232-6 (PMC10203120; doi:10.1038/s41598-023-34232-6)
Supplement: Supplementary file 1 — Supplementary Information. [file 41598_2023_34232_MOESM1_ESM.pdf]

Supplementary material

Title: Seg2Link: an efficient and versatile solution for semi-automatic cell segmentation in 3D image stacks

Authors:

Chentao Wen[1, 2*], Mami Matsumoto[3,4], Masato Sawada[3,4], Kazunobu Sawamoto[3,4], Koutarou D Kimura[1]

1. Graduate School of Science, Nagoya City University, Nagoya, Japan

2. RIKEN Center for Biosystems Dynamics Research, Kobe, Japan

3. Department of Developmental and Regenerative Neurobiology, Institute of Brain Science, Nagoya City University Graduate School of Medical Sciences, Nagoya, Japan

4. Division of Neural Development and Regeneration, National Institute for Physiological Sciences, Okazaki, Japan

* chentao.wen@riken.jp

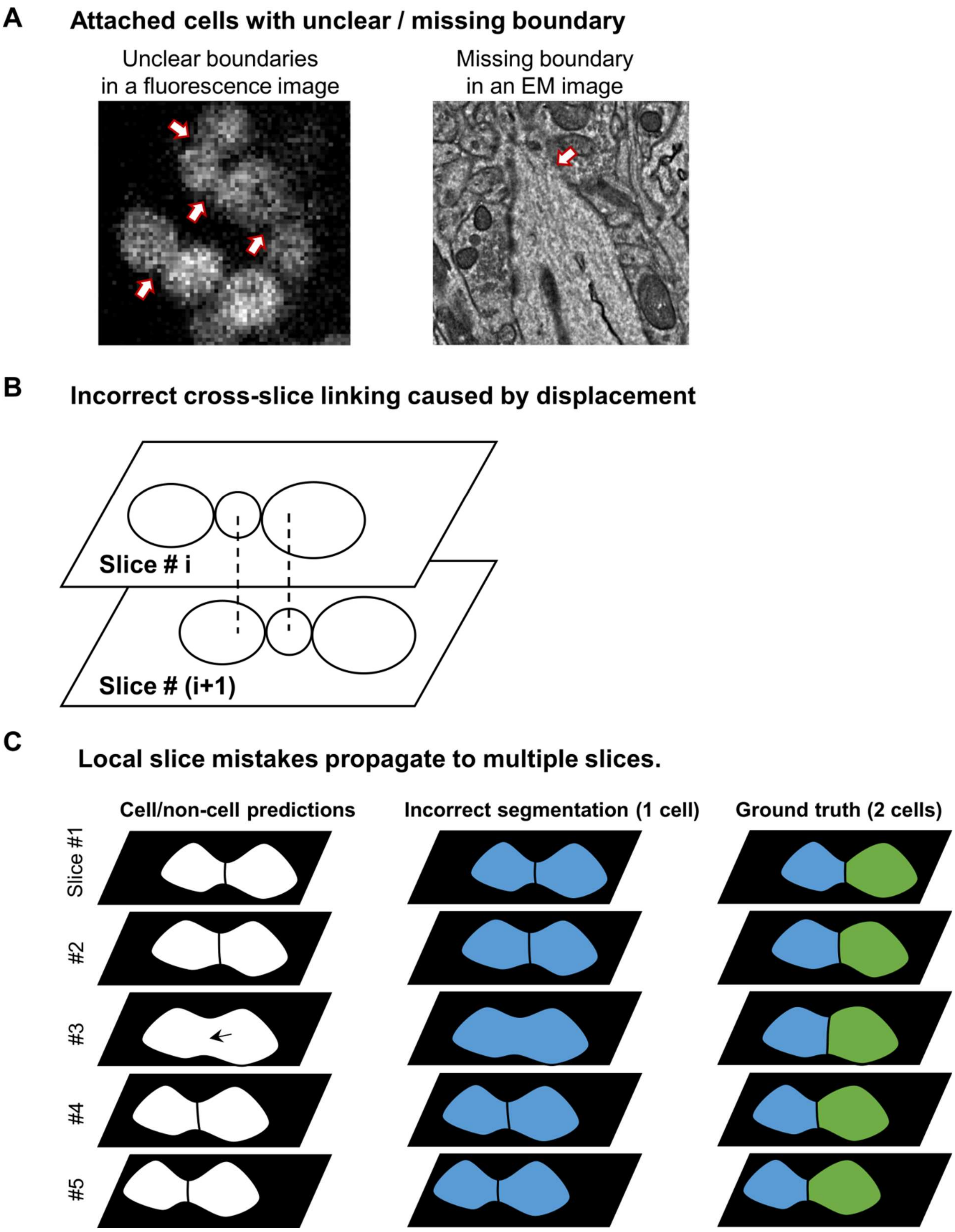

Supplementary Figure S1. The illustrations of the challenges encountered in automatic instance segmentation. (A) The challenges in discerning cell boundaries in real images, indicated by red arrows,

which can result in incorrect segmentation results. These two example images are identical to those in Fig. 5. (B) An illustration of how displacement across neighboring slices can cause incorrect cell linking. (C) An illustration of the local mistakes (arrow) propagated to multiple slices.

A

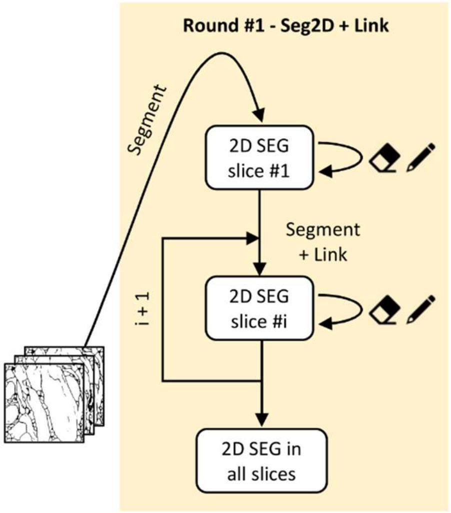

B

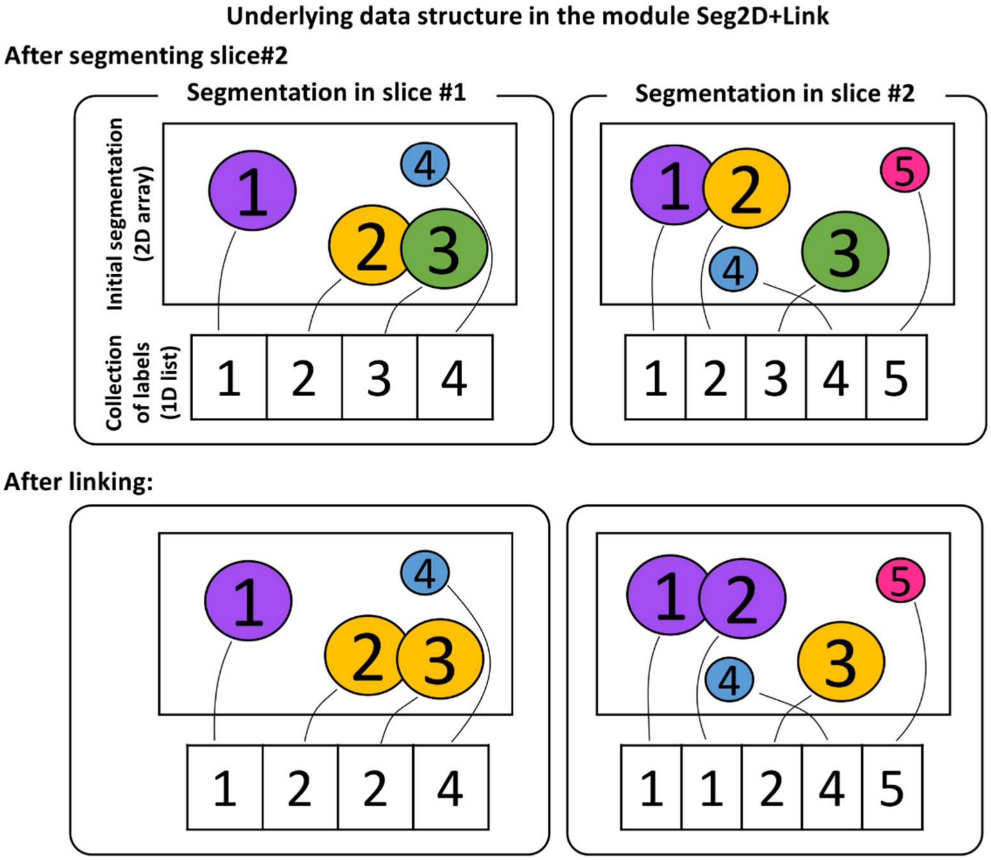

Supplementary Figure S2. The underlying data structure of the Seg2D+Link module. (A) The workflow in the Seg2D+Link module. (B) A diagram of the underlying data structure, as well as the overlap linking process based on the data structure.

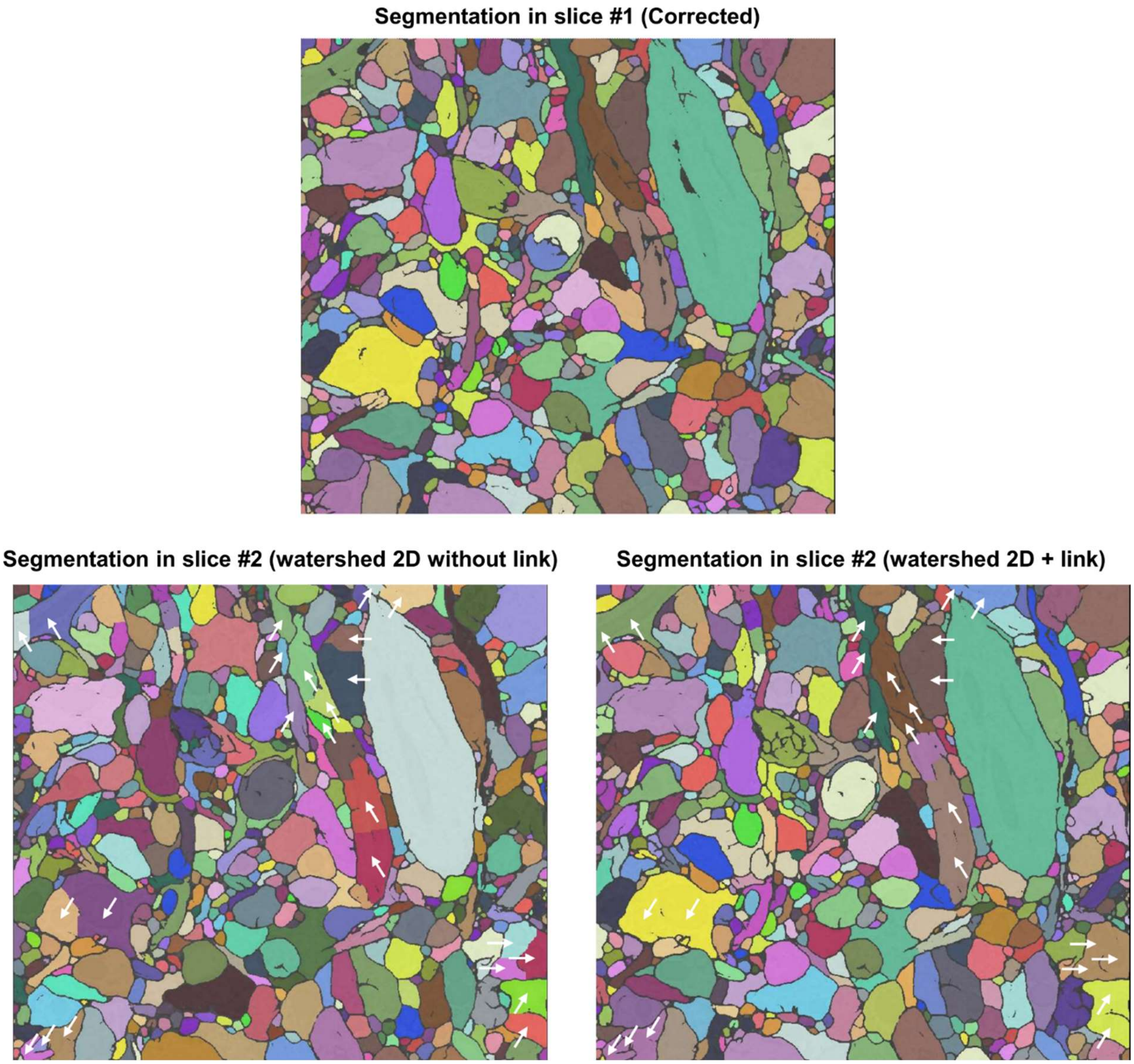

Supplementary Figure S3. Corrections made in a previous slice can improve automatic segmentation in subsequent slices. (Top) Manually corrected segmentation in slice #1. (Bottom left) Automatic segmentation in slice #2 using watershed 2D but no link. (Bottom right) Automatic segmentation in slice #2 using watershed 2D + link. The arrows point to the same regions that were split incorrectly by watershed 2D and correctly merged by watershed 2D + link. Colors were automatically assigned by napari.

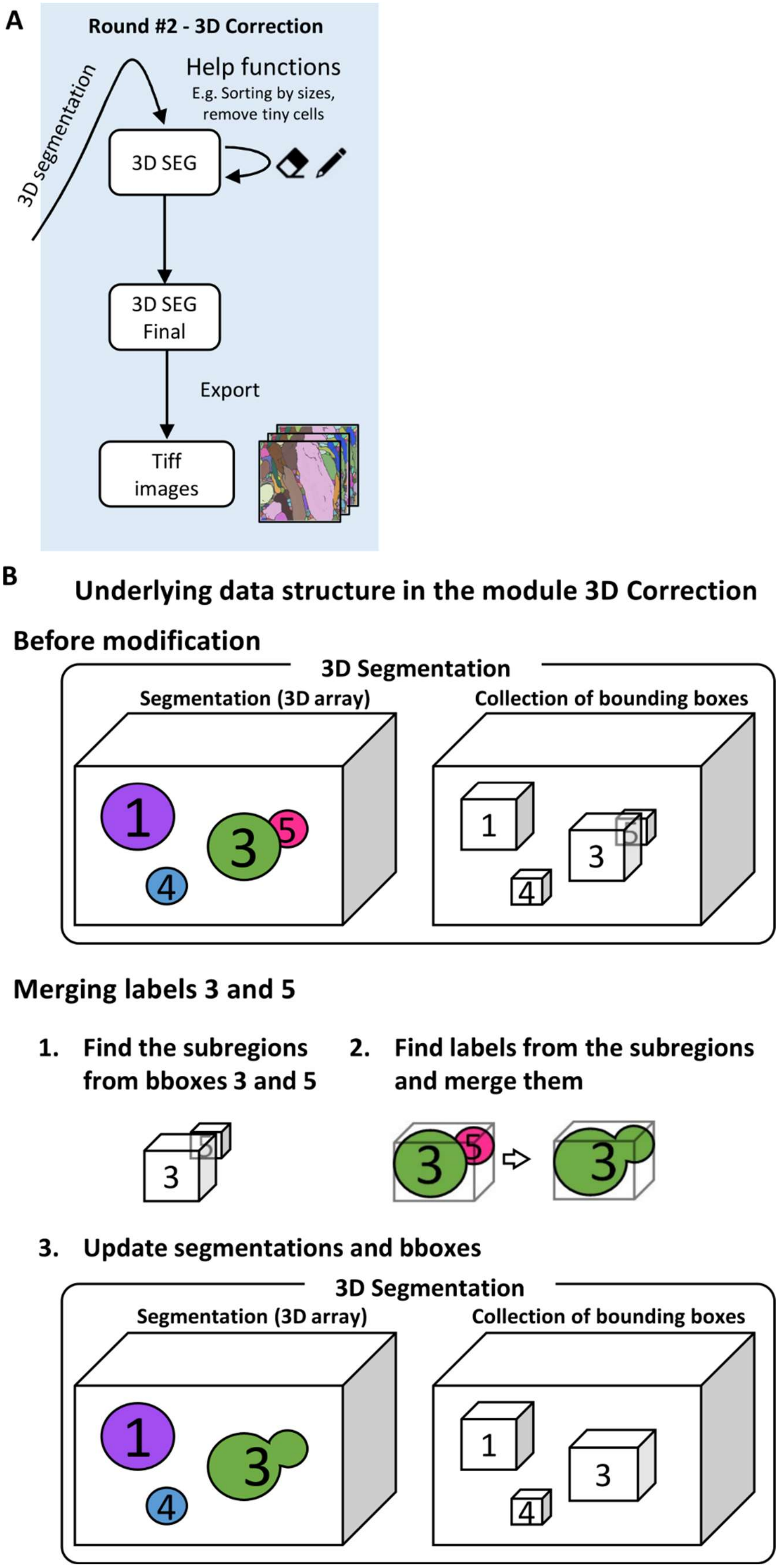

A
Round #2 - 3D Correction
3D segmentation
Help functions
E.g. Sorting by sizes,
remove tiny cells
3D SEG
3D SEG
Final
Export
Tiff
images
B
Underlying data structure in the module 3D Correction
Before modification
3D Segmentation
Segmentation (3D array)
Collection of bounding boxes
1
3
5
4
1
3
4
Merging labels 3 and 5
1. Find the subregions from bboxes 3 and 5
2. Find labels from the subregions and merge them
3
3
5
3
3. Update segmentations and bboxes
3D Segmentation
Segmentation (3D array)
Collection of bounding boxes
1
3
4
1
3
4

Supplementary Figure S4. The underlying data structure of the 3D correction module. (A) The workflow in the 3D correction module. (B) A diagram of the underlying data structure, as well as the merge process based on the data structure.

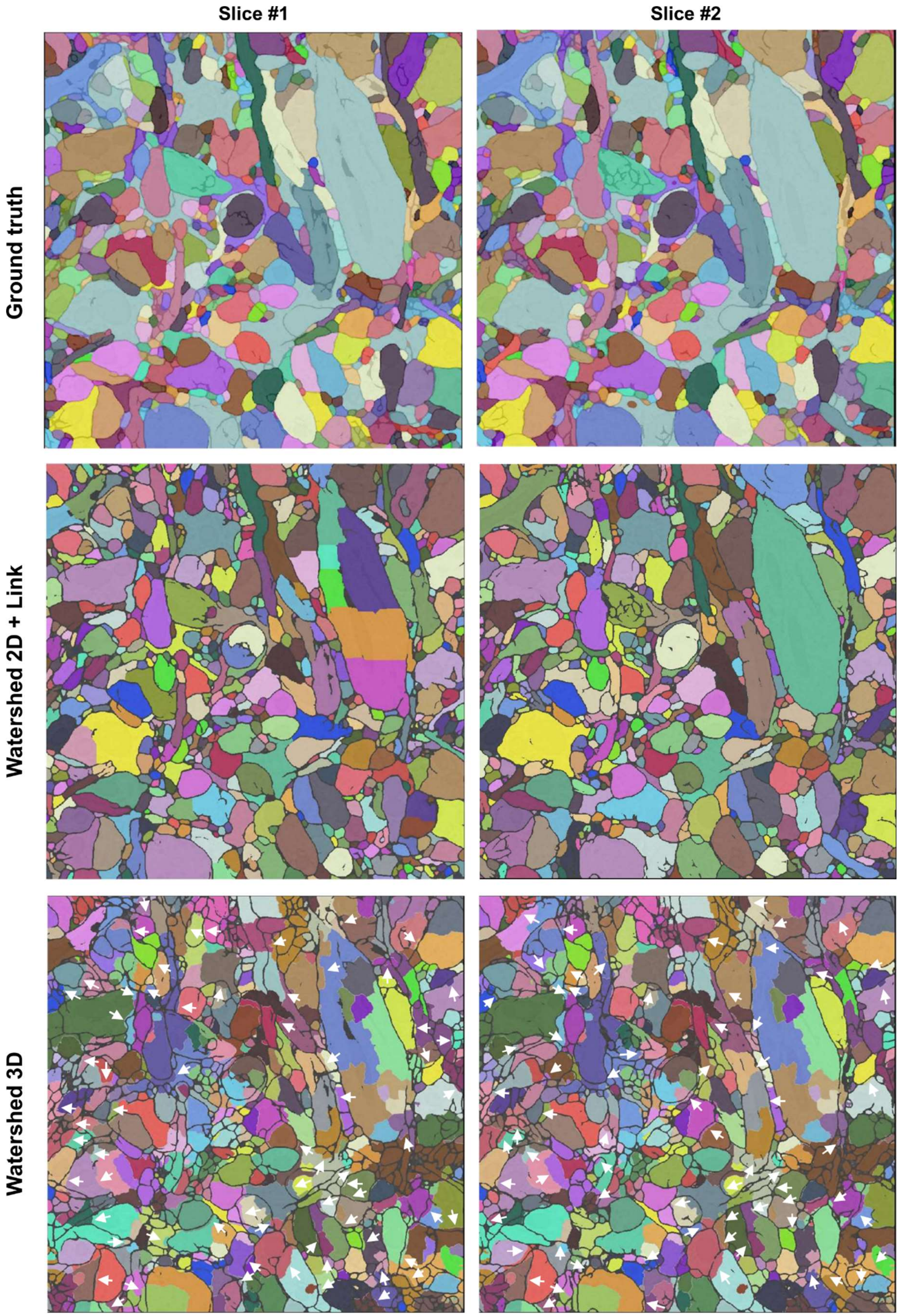

Slice #1
Slice #2
Ground truth
Watershed 2D + Link
Watershed 3D

Supplementary Figure S5. Comparison of the boundary mistakes found in the segmentation results of two slices in the EM demo dataset, obtained by watershed 2D + Link method and watershed 3D method. The Ground Truth is also shown as a reference. The arrows indicated the boundary mistakes identified by visual inspection. It is worth noting that only mistakes resulting from the watershed 2D /3D were considered, while those caused by incorrect deep learning predictions were disregarded. We did not find such boundary mistake in the watershed 2D + Link results.

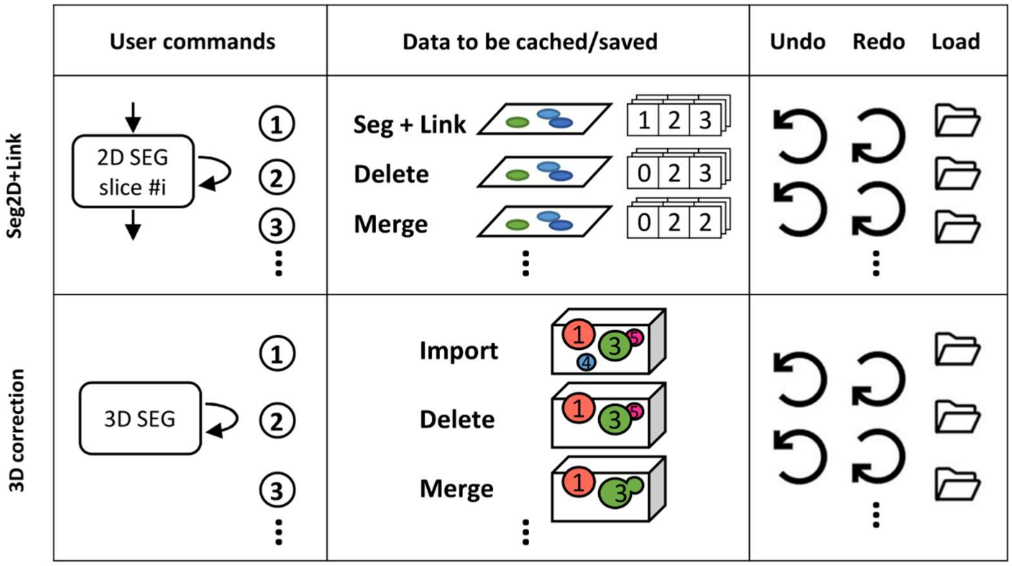

Supplementary Figure S6. The caching/saving methods used in the Seg2D+Link and 3D correction modules. The data to be cached/saved in the two modules after performing each user command. Note that in reality, our 3D correction module only caches a subregion of the entire 3D array to reduce the memory utilization.

Supplementary Table S1. Operations that alter elements of the data structure of the Seg2D+Link module. c.w.s: current working slice.

Operations	Elements affected by the operation
Next slice (segment, segment + link)	2D segmentation (c.w.s) + Label lists
Division/Division-Relink	2D segmentation (c.w.s) + Label lists
Cache/Save intermediate state	2D segmentation (c.w.s) + Label lists
Merge	Label lists
Delete	Label lists

Supplementary Table S2. A comparison of the two modules' efficiency in saving a real data (EM demo dataset). Our software saves each 2D array in npz format (compressed), label lists in pickle format, and 3D arrays in npy format (without compression since it's time-consuming for large data). The time is estimated assuming the write speed of the hard disk is 100 MB/sec. c.w.s: current working slice.

Module	Data to be saved	Data size	Time for saving
Seg2D+Link	2D array (c.w.s)<br>+ label lists (1 – c.w.s)	**Total:** 82 KB ~ 2.0 MB<br>**-** 2D array: 80 KB / slice<br>- Label list: 1.61 KB / slice	0.8 ~ 20 ms
3D correction	3D array	**Total:** 2.46 GB	25 sec
